# Inhibition of Poly(ADP-Ribose) Polymerase by Nucleic Acid Metabolite 7-Methylguanine

**Published:** 2016

**Authors:** D. K. Nilov, V. I. Tararov, A. V. Kulikov, A. L. Zakharenko, I. V. Gushchina, S. N. Mikhailov, O. I. Lavrik, V. K. Švedas

**Affiliations:** Lomonosov Moscow State University, Belozersky Institute of Physicochemical Biology, Leninskie Gory 1, bldg. 40, Moscow, 119991, Russia; Lomonosov Moscow State University, Faculty of Bioengineering and Bioinformatics, Leninskie Gory 1, bldg. 73, Moscow, 119991, Russia; Engelhardt Institute of Molecular Biology, Russian Academy of Sciences, Vavilov str. 32, Moscow, 119991 , Russia; Lomonosov Moscow State University, Faculty of Fundamental Medicine, Lomonosovsky avenue 31 -5, Moscow, 119192, Russia; Institute of Chemical Biology and Fundamental Medicine, Russian Academy of Sciences, Siberian Branch, Lavrentiev avenue 8, Novosibirsk, 630090, Russia

**Keywords:** PARP inhibitors, molecular modeling, docking

## Abstract

The ability of 7-methylguanine, a nucleic acid metabolite, to inhibit poly(ADP-ribose)polymerase-1 (PARP-1) and poly(ADP-ribose)polymerase-2 (PARP-2) has been identified *in silico *and studied experimentally. The amino group at position 2 and the methyl group at position 7 were shown to be important substituents for the efficient binding of purine derivatives to PARPs. The activity of both tested enzymes, PARP-1 and PARP-2, was suppressed by 7-methylguanine with IC_50_ values of 150 and 50 μM, respectively. At the PARP inhibitory concentration, 7-methylguanine itself was not cytotoxic, but it was able to accelerate apoptotic death of BRCA1-deficient breast cancer cells induced by cisplatin and doxorubicin, the widely used DNA-damaging chemotherapeutic agents. 7-Methylguanine possesses attractive predictable pharmacokinetics and an adverse-effect profile and may be considered as a new additive to chemotherapeutic treatment.

## INTRODUCTION

Exposure of a human organism to different stress factors induces genotoxic DNA lesions that should be removed in order to ensure complete and accurate DNA replication and transcription, to avoid genomic instability, and to prevent, for example, cancer formation. Cellular repair pathways involve numerous proteins that recognize and clear DNA base modifications and DNA strand breaks [1]. Poly(ADP-ribose)polymerases (PARP; EC 2.4.2.30) are a group of eukaryotic proteins with diverse functions mainly related to DNA repair and cell death. The most studied PARP family members, PARP-1 and PARP-2, have DNA-damage-dependent enzymatic activity and catalyze the synthesis of poly(ADP-ribose) [2]. The donor of the ADP-ribose unit in the polymer synthesis is the NAD^+^ molecule, and nicotinamide is released while a glycosidic bond between the units is formed. Binding of the PARP-1 and PARP-2 proteins to damaged DNA results in their poly(ADP-ribosyl)ation and that of the other proteins involved in DNA metabolism [3-6]. This kind of posttranslational modification leads to the activation and assembly of repair systems in the damaged locus of DNA: for example, automodified PARP-1 recruits the base excision repair protein XRCC1 associated with DNA polymerase *β *and DNA ligase III [7-9]. The crucial role of PARP-1 and PARP-2 has been demonstrated by observations that both *parp-1-/- *and *parp- 2-/- *mice are more sensitive to ionizing radiation, and* parp-1^-/-^parp-2^-/-^*double mutants die early in development at the onset of gastrulation [10].

The DNA-binding domain (DBD) of PARP-1 is made of specialized zinc fingers, whereas the DBD structure of PARP-2 is unknown and has no sequence homology with any identified DNA-binding motif. In contrast, the catalytic domains and the active sites of PARP-1 and PARP-2 in the apo form, as well as in a complex with inhibitors, have extensive structural similarity [11,12]. The NAD^+^ substrate bound in the active site interacts with Gly863 and Tyr907 residues (the numeration is for PARP-1) similar to inhibitors that mimic nicotinamide moiety. The Gly863 backbone forms two hydrogen bonds with the amide group of nicotinamide, while the Tyr907 side chain stacks with the nicotinamide ring [13]. Several known classes of PARP inhibitors are composed of a carbamoyl group attached to an aromatic ring or a lactam group built in an aromatic ring system [14-19], which makes possible the formation of the abovementioned interactions with the Gly863 and Tyr907 residues. Besides compounds competing with NAD^+^ for the active site, the minor groove binding ligands may also serve as inhibitors that target the DNA-dependent pathway of PARP-1 regulation [20].

The PARP’s involvement in DNA repair systems makes this enzyme an attractive target for anticancer therapy. Inhibitors of PARP-1 and PARP-2 may potentiate the effects of various DNA-damaging anticancer drugs, such as cisplatin or doxorubicin. When DNA is moderately damaged, PARPs participate in DNA repair so that cancer cells can survive. The combination of a DNA-damaging agent and PARP-1 or PARP-2 inhibitors can help to overcome drug resistance and promote apoptotic cell death, representing a promising strategy for cancer treatment [15, 21-23]. In addition, the use of inhibitors can exploit DNA repair defects in certain cancer cells. For example, the deficiency in homologous recombination in BRCA1/2-deficient cells makes them acutely sensitive to PARP inhibition [24-26]. Several PARP inhibitors tested as anticancer agents have failed to progress through preclinical or clinical trials because of their toxicity and insufficient efficacy [27-29]. In particular, a well-known PARP-1 inhibitor, 3-aminobenzamide, has a limited cell uptake and affects other metabolic processes. A first-in-class PARP-1 inhibitor, olaparib, was approved by the FDA in December 2014 as treatment for patients with advanced ovarian cancer [30]. This compound is a phthalazine derivative with a lactam group which decreases the enzyme’s activity at a nanomolar concentration. Nevertheless, developing effective and non-toxic compounds targeting PARPs and able to suppress the progression of various types of cancers is an important, yet challenging task.

One of the promising classes of PARP inhibitors comprises natural nucleobases and their derivatives which contain a lactam group [31, 32]. However, so far identified compounds (e.g., thymine, hypoxanthine) exert a relatively weak inhibitory effect. In this paper, we report on the results of a computer screening of nucleobase derivatives as PARP inhibitors and *in vitro* studies of the selected compounds.

## EXPERIMENTAL SECTION

**Protein model preparation**

The initial model of PARP-1 was built on the basis of the 1efy crystallographic structure of the enzyme complex with inhibitor [33] using the AmberTools 1.2 program package (http://ambermd.org). Hydrogen atoms were added to the protein structure, and then it was solvated by a 12 A-thick layer of TIP3P water. Chloride ions were added to neutralize the system. To perform the energy minimization of the obtained model, the protein molecule was described by the *ff99SB *force field [34] and the inhibitor molecule was described by* GAFF *parameters [35] calculated automatically. The energy minimization (2,500 steps of the steepest descent algorithm followed by 2,500 steps of the conjugate gradient algorithm) was performed using the Amber 10 package [36] in order to optimize the positions of hydrogen atoms. During the minimization, the heavy atoms of the protein and inhibitor were kept fixed by positional restraints *k*(Δ*x*)2, where the force constant* k *was 2 kcal/(mol A2). The inhibitor, water molecules, and chloride ions were removed from the system after the energy minimization to obtain a model for molecular docking.

**Molecular docking**

The computer library of natural nucleobase derivatives was prepared with the ACD/ChemSketch program [37]. Molecular docking was performed using the Lead Finder 1.1.14 program [38]. The energy grid map surrounding the active site of the PARP-1 model was calculated, and the library was screened using the genetic search algorithm. A series of 20 independent docking runs was performed for each compound, and the probability of a successful docking *P*_dock_ was defined as the ratio of the number of successful runs meeting the specified structural criterion to the total number of runs; i.e., *P*_dock_ = *N*_succ_/20. The structural criterion was the presence of two hydrogen bonds between the lactam group of a docked compound and the Gly863 residue. Compounds with *P*_dock_ ≤ 0.8 were sorted out automatically by a Perl script.

**Molecular dynamics simulation**

To include the selected potential inhibitor in the simulation, its parameters, except partial charges, were taken from the *ff99SB *force field. To derive partial charges, the molecular electrostatic potential of the inhibitor was calculated at the HF/6-31G* level of theory with the PC GAMESS/Firefly program [39]. The fitting of partial atomic charges was done using the RESP method [40]. An equilibration and subsequent 10 ns molecular dynamics (MD) simulation of the PARP-1 in complex with the inhibitor were carried out using AmberTools 1.2 and Amber 10. A model of the complex obtained by molecular docking was solvated by a 12 A-thick layer of TIP3P water and described by the* ff99SB *force field. The energy minimization using the steepest descent and conjugate gradient algorithms was performed to relax the solvated system. The minimized system was heated up from 0 to 300 K over 50 ps and then equilibrated over 500 ps at 300 K. Finally, a 10 ns trajectory of an equilibrium simulation at constant pressure was calculated. All simulations were performed using periodic boundaries and the Particle Mesh Ewald method to calculate long-range electrostatic interactions.

The VMD 1.8.6 software [41] was used for the visualization of the structures. Parallel computations of the MD trajectory were performed at the Supercomputer Center, Lomonosov Moscow State University [42].

**Synthesis of compounds**

7-Methylguanine, 7-methylxanthine, 7-methylhypoxanthine, and 7-ethylguanine were prepared by alkylation of the corresponding nucleosides, followed by N-glycosidic bond cleavage according to the earlier described procedures [43,44].

*7-Methylguanine. *400 MHz 1H NMR (DMSO-d6): δ = 3.82 (s, 3H, Me), 6.03 (brs, 2H, NH2), 7.81 (s, 1H, H-8), 10.66 (brs, 1H, NH).

*7-Methylxanthine. *400 MHz ^1^H NMR (DMSO-d6): δ = 3.81 (s, 3H, Me), 7.85 (s, 1H, H-8), 10.79 (brs, 1H, NH), 11.48 (brs, 1H, NH).

*7-Methylhypoxanthine. *400 MHz ^1^H NMR (CD-Cl_3_-CD_3_OD): δ = 3.94 (s, 3H, Me), 7.80 (s, 1H, H-2), 7.84 (s, 1H, H-8).

*7-Ethylguanine. *400 MHz ^1^H NMR (DMSO-d_6_): δ = 1.36 (t, 3H, J = 7.2 Hz, CH_3_), 4.19 (q, 2H, Me, J = 7.2 Hz, CH_2_), 6.09 (brs, 2H, NH_2_), 7.90 (s, 1H, H-8), 10.26 (brs, 1H, NH).

**Enzyme assay**

Recombinant human PARP-1 and murine PARP- 2 proteins were purified as described previously [45, 46]. Reaction of poly(ADP-ribosyl)ation catalyzed by PARP-1 and PARP-2 was performed at optimal conditions for each enzyme [47,48]. Briefly, for PARP-1: 50 mM Tris-HCl pH 8.0, 20 mM MgCl_2_, 150 mM NaCl, 7 mM β-mercaptoethanol, activated DNA (2 o.u._280_/ml, degree of activation 25%), 300 •µM NAD^+^ (0.18 •µCi [^3^H]NAD^+^), 37°C. The reaction was started by adding PARP-1 up to a final concentration of 0.2 μM and was stopped after 1 min, placing the reaction mixture on paper filters (Whatman-1) soaked with a 5% solution of trichloroacetic acid. For PARP-2: 50 mM Tris-HCl pH 8.0, 40 mM NaCl, 0.1 mg/ml BSA, 8 mM MgCl_2_, 1 mM DTT, activated DNA (2 o.u._280_/ml, degree of activation 25%), 400 •µM NAD^+^ (0.4 •µCi [^3^H]NAD^+^), 37°C. The reaction was started by adding PARP-2 up to a final concentration of 0.2 μM and was stopped after 5 min, placing the reaction mixture on the paper filters. The filters were washed four times by the 5% trichloroacetic acid, then by 90% ethanol (to remove acid), and air-dried. The quantity of radiolabel included into the acid insoluble product was registered on a scintillation counter Tri-Carb 2800 (Perkin Elmer) in a toluene scintillator. The quantity of the radiolabeled product was determined at the initial rate period.

The PARP-inhibiting activity of the synthesized compounds was evaluated in a reaction of auto- poly(ADP-ribosyl)ation at a NAD^+^ concentration of 0.3 mM for PARP-1 and 0.4 mM for PARP-2. Different concentrations of the tested compounds were added to the reaction mixture before adding the enzyme. Reaction and detection of the products were performed as described above. To determine the IC_50_ value (concentration of the compound required to reduce the enzyme activity by 50%), the effect of different concentrations of the inhibitor on the enzyme activity was examined. Measurements were done in at least two independent experiments. IC_50_ values were calculated using the Origin Pro 8.0 software by nonlinear regression analysis.

**Cytotoxicity assay**

The cytotoxic activity of 7-methylguanine, cisplatin, doxorubicin, and their combinations was evaluated by the analysis of cell cycle distribution and measurement of the Sub-G1 population by flow cytometry, as well as by measurement of caspase-3-like activity as a marker of the apoptotic pathway. A BRCA1-deficient human breast cancer line HCC1937 (ATCC CRL-2336) was cultured in DMEM supplemented with 10% heat-inactivated fetal bovine serum, penicillin/streptomycin (100 U/ml), and pyruvate (0,11 mg/ml) at 37°C in 20% O_2_ humidified atmosphere. The cells were maintained in a logarithmic growth phase for all experiments. After 24 h of culturing, the cells were pretreated with 7-methylguanine (150 µM) for 3 h, followed by addition of either cisplatin (70 µM) or doxorubicin (1 µM).

To perform cell cycle analysis, the cells were then harvested after 72 hours, fixed with 70% EtOH (final concentration) for 60 min on ice, rinsed in PBS, and stained in a 500 •µl solution containing 50 •µg/ml propidium iodide and 25 •µg/ml RNase A for 15 min. Data were acquired by a BD FACS CantoII flow cytometer (BD Biosciences) and analyzed using the FACSDiva software. The cleavage of the fluorogenic peptide substrate Ac-DNLDAMC was measured using a fluorometric assay. Upon treatment with cytotoxic agents, the cells were incubated for 48 hours, then harvested and washed with PBS. After centrifugation, they were re-suspended in PBS at a concentration of 2 × 10^6^ cells/100 μl. Then, 25 μl of the suspension was added to a 96-well plate and mixed with a DEVD peptide substrate dissolved in a standard reaction buffer (100 mM HEPES, 10% sucrose, 5 mM DTT, 0.001% NP- 40, and 0.1% CHAPS, pH 7.2). Cleavage of the fluorogenic peptide substrate was monitored by AMC liberation in a VarioScan Flash multimode detector (Thermo Scientific) using 380 nm excitation and 460 nm emission wavelengths. Measurements were done in at least two independent experiments.

**Pharmacokinetics and adverse-effect modeling**

Pharmacokinetics and adverse-effect profiling of 7-methylguanine was done with ACD/Percepta [49]. This software *in silico *predicts ADME properties (absorption, distribution, metabolism, excretion) and toxicity by QSAR models based on an analysis of similar compounds from the experimental data library. In case of 7-methylguanine, among library compounds were acyclovir, caffeine, theobromine, and theophylline.

## RESULTS AND DISCUSSION

**Virtual screening**

A model of PARP-1, the most characterized member of the PARP family, was built on the basis of the crystallographic structure of the catalytic fragment in a complex with the inhibitor (PDB ID 1efy, 2.2 A resolution). Hydrogen atoms were added taking into account ionization of amino acid side chains, and then their positions were optimized to achieve complementarity to the inhibitor scaffold. A computer library of natural nucleobase derivatives with a lactam structural fragment was prepared comprising nearly a hundred diverse purine and pyrimidine modifications which could be synthesized on a preparative scale. Virtual screening for derivatives able to bind in the active site of the PARP-1 was performed by molecular docking. In order to provide a better sampling of the conformational space, a series of 20 independent docking runs was performed for each compound in the library. Then, we applied the procedure of structural filtration, which allows one to sort out false-positive docking results [47]. As it has been noticed previously, the substrate and the known PARP inhibitors have a common structural feature – their amide (or lactam) group forms two hydrogen bonds with the Gly863 residue. This interaction is apparently crucial for an effective binding in the PARP active site and was used as a criterion for selection of potential inhibitors. Docking poses of compounds meeting the structural criterion were further analyzed for favorable hydrophobic contacts as well as electrostatic interactions in the PARP-1 active site, and the 7-methylguanine molecule (*P*_dock_ = 0.95, Δ*G*_calc_ = –6.8 kcal/mol) was selected as the most promising PARP inhibitor.

**Fig. 1:**
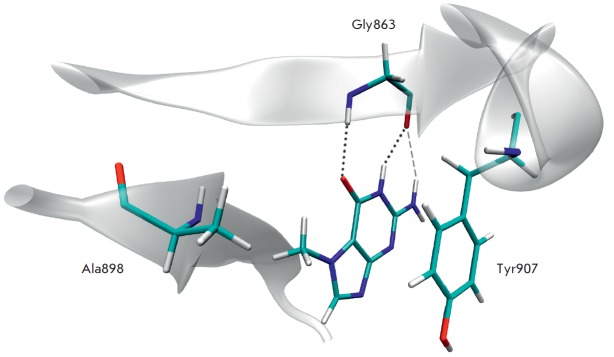
The position and interactions of the 7-methylguanine molecule in the PARP-1 active site revealed by molecular modeling: two hydrogen bonds of the lactam group with Gly863 shown as dotted lines, an electrostatic interaction of the amino group as dashed line, pi stacking of purine rings with Tyr907, and hydrophobic interaction of the methyl group with Ala898.

MD simulations were further performed to evaluate the geometric characteristics of 7-methylguanine in the PARP-1 active site and examine the stability of the enzyme-inhibitor complex. The formation of two hydrogen bonds between the lactam group of 7-methylguanine and the Gly863 residue was observed along the MD trajectories as well as the pi stacking of purine rings with the side chain of Tyr907 and the hydrophobic interaction of the methyl group at position 7 with the Ala898 side chain (*Fig. 1*). We also revealed an electrostatic interaction between the amino group of 7-methylguanine at position 2 and the backbone oxygen of Gly263, which appeared to be a non-conventional hydrogen bond. The mean NH2:H∙∙∙Gly863:O distance was 2.42 A, and the mean NH2:N∙∙∙NH2:H∙∙∙Gly863:O angle 137°, whereas the corresponding distance of a regular hydrogen bond is expected to be 1.8–2.1 A and the angle not less than 150°. Distance and angle characteristics are presented in *Table 1*.

**Table 1:** Distance and angle characteristics of the position of 7-methylguanine (7-MG) in the PARP-1 active site determined by MD simulations. Mean values are presented together with the standard deviations.

Distance (Å)	
7-MG:CO:O ∙∙∙ Gly863:H	2.0 ± 0.2
7-MG:NH:H ∙∙∙ Gly863:O	1.9 ± 0.1
7-MG:NH_2_:H ∙∙∙ Gly863:O	2.4 ± 0.4
7-MG:CH_3_:C ∙∙∙ Ala898:CB	4.0 ± 0.3
C(7-MG fused rings) ∙∙∙ C(Tyr907 benzene ring)^*^	3.6 ± 0.2
Angle (deg)	
7-MG:CO:O ∙∙∙ Gly863:H ∙∙∙ Gly863:N	160 ± 11
7-MG:NH:N ∙∙∙ 7-MG:NH:H ∙∙∙ Gly863:O	159 ± 9
7-MG:NH_2_:N ∙∙∙ 7-MG:NH:H ∙∙∙ Gly863:O	137 ± 10

^*^Distance between the geometric center of 7-methylguanine fused rings and the center of the Tyr907 benzene ring.

**Fig. 2:**
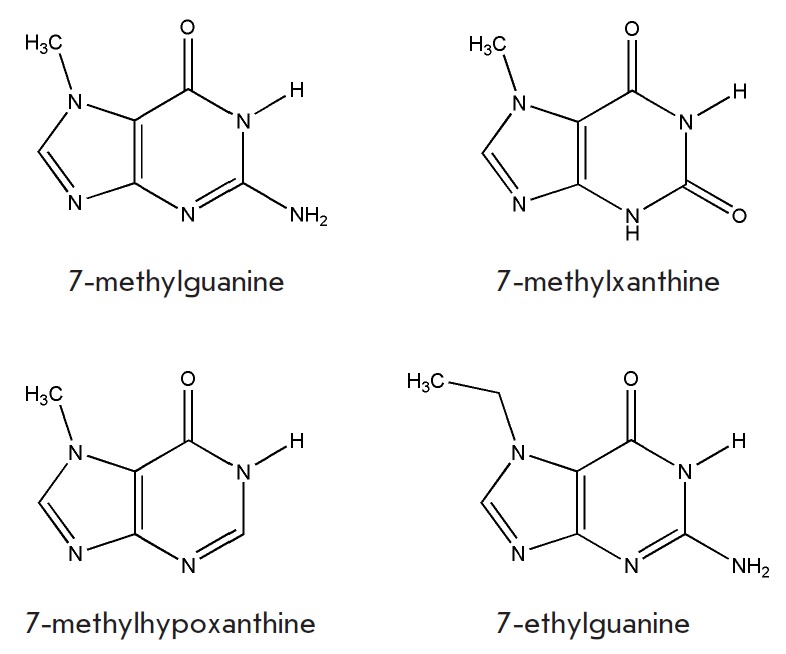
Chemical structures of the synthesized and tested compounds.

Interestingly, the structural analogue of 7-methylguanine, namely, 7-methylxanthine, was previously shown to be a moderate inhibitor of PARP-1 [32]. This compound differs from 7-methylguanine by an oxo substituent at position 2 (*Fig. 2*). However, 7-methylxanthine was sorted out by our procedure of structural filtration (*P*_dock_ = 0.45), indicating that its binding has to be less effective. We also docked 7-methylhypoxanthine, analogue with no substituent at position 2, and the predicted binding parameters (*P*_dock_ = 0.85, Δ*G*_calc_ = –6.4 kcal/mol) were less encouraging, as well. Analysis of the modeled poses demonstrated that the amino group at position 2 can substantially increase the effectiveness of the inhibitor’s binding in the PARP active site due to the favorable electrostatic interaction with Gly863. The methyl group at position 7 is another substituent responsible for the complementarity of the inhibitor to the PARP-1 active site, as the unmodified xanthine does not show inhibition [32]. However, the calculated parameters of 7-ethylguanine binding (*P*_dock_ = 0.7, Δ*G*_calc_ = –.7 kcal/mol) indicate that the inhibitory effect cannot be further increased with a growing alkyl chain at this position.

**Inhibitory properties of purine derivatives**

We synthesized 7-methylguanine, 7-methylxanthine, 7-methylhypoxanthine, and 7-ethylguanine to test their ability to suppress PARP and assess the effect of the substituent on the activity of the inhibitor. The inhibitory properties of 7-methylguanine and related compounds were studied using two purified proteins of the PARP family – human PARP-1 and murine PARP-2. Experimental data presented in *Table 2* demonstrate that 7-methylguanine, as predicted, is the most effective inhibitor, with IC_50_ values of 150 and 50 μM for PARP-1 and PARP-2, respectively. Replacement of the 2-oxo group of 7-methylxanthine by the amino group led to a 5- and 3-fold increase in the ability to inhibit PARP-1 and PARP-2. 7-Methylguanine was a more effective inhibitor compared to 7-ethylguanine, indicating that the methyl group is an optimal alkyl substituent at this position. It is worth mentioning that all tested purine derivatives were more effective inhibitors of PARP-2 despite the very similar organization of the binding sites of both enzymes. We can assume that the reason for this selectivity is the different inhibitor delivery trajectories to the active centers of the PARP proteins.

**Table 2:** Inhibitory effect of 7-methylguanine and related compounds on PARP-1 and PARP-2.

	IC_50_ (μM)	
	PARP-1	PARP-2
7-methylguanine	150	50
7-methylxanthine	800	160
7-methylhypoxanthine	780	620
7-ethylguanine	230	90

**Analysis of cytotoxicity**

**Fig. 3:**
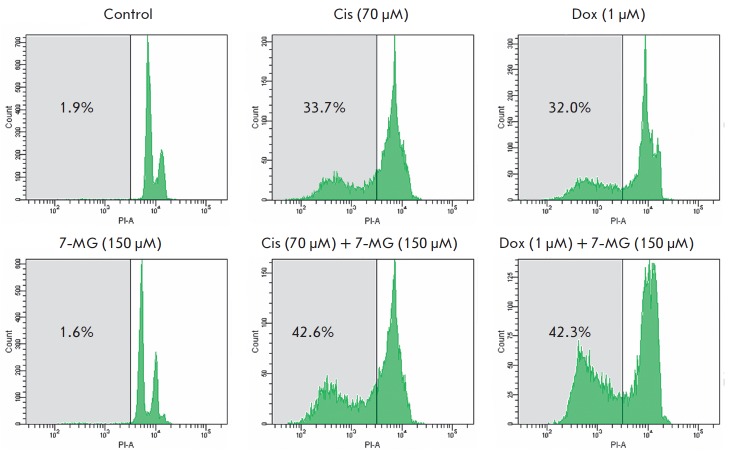
Estimation of the Sub-G1 population of HCC1937 cells subjected to cisplatin (Cis), doxorubicin (Dox), and 7-methylguanine (7-MG) in single and combined treatment for 72 h. The area of the Sub-G1 population is shown in gray.

Analysis of cytotoxicity was performed on a human breast cancer line HCC1937, which is thought to be sensitive to the inhibition of PARP due to deficiency in the DNA repair gene BRCA1 [22, 50, 51]. Cell death induced by the conventional anticancer drugs cisplatin and doxorubicin and by 7-methylguanine was estimated by flow cytometry analysis of a Sub-G1 population, which corresponds to an apoptotic cell population with fragmented DNA (*Fig. 3*). Treatment of the cells with 7-methylguanine itself did not increase the cells’ number in the Sub-G1 phase (it was around 2%), which was comparable to the control. Comparison of cell death level revealed that 7-methylguanine sensitizes HCC1937 to treatment with cisplatin and doxorubicin. With the exposure of cells to a combination of 7-methylguanine and 70 •µ M cisplatin, the population of cells in the Sub-G1 phase increased from 34% to 43% and addition of 7-methylguanine to 1 •µ M doxorubicin increased the Sub-G1 population from 32% to 42%. Thus, the level of cell death elevation at addition of 7-methylguanine was very similar in the cases of cisplatin and doxorubicin.

**Fig. 4:**
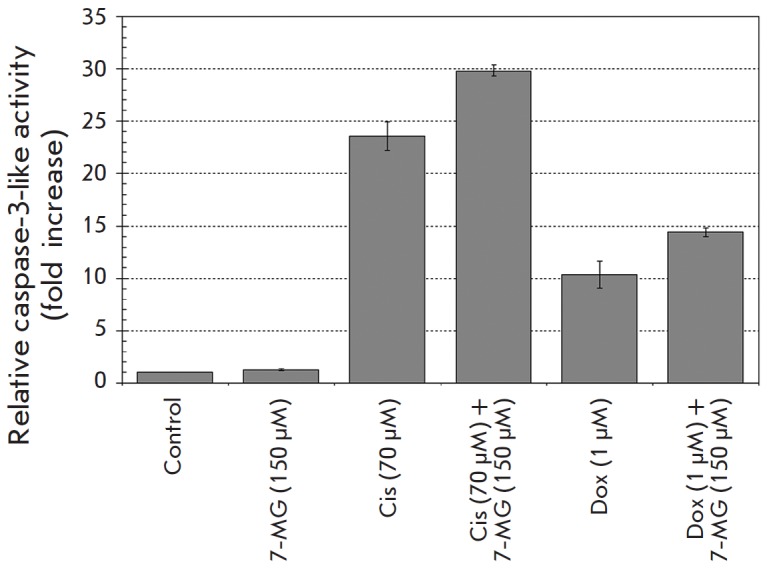
Estimation of caspase-3-like activity in HCC1937 cells subjected to cisplatin (Cis), doxorubicin (Dox), and 7-methylguanine (7-MG) in single and combined treatment for 48 h.

We also analyzed the activation of caspase-3 in HCC1937 cells, which is an important and obligatory event in the apoptotic cell death program. Active caspase-3 cleaves various cellular molecules, which results in apoptotic morphology of cells. Thus, the degree of caspase-3 activation, measured by cleavage of the specific fluorogenic substrate, corresponds to the level of apoptotic cell death. *Figure 4* demonstrates that stimulation of caspase-3 activity was increased by the addition of 7-methylguanine to either cisplatin or doxorubicin by 27–39%, whereas 7-methylguanine alone demonstrated no caspase-3 activation. These data are in agreement with cell death induction observed by flow cytometry.

**Pharmacokinetics and adverse-effect profiling**

Finally, we evaluated the pharmacokinetic properties and adverse-effect profile of 7-methylguanine using QSAR models based on literature data on its structural analogues (acyclovir, caffeine, theobromine, theophylline, etc.). In particular, human intestinal permeability was estimated to be very high, and the oral bioavailability was predicted to be optimal (83%). The calculated plasma protein bound fraction of 7-methylguanine was 17%, which should not considerably affect its efficiency. It is unlikely that 7-methylguanine binds to estrogen receptor alpha (no risk of reproductive toxicity), hERG potassium ion channel (no risk of cardiotoxicity), P-glycoprotein efflux transporter, and cytochrome P450 enzymes (CYP3A4, CYP2D6, CYP2C9, CYP2C19, CYP1A2). Thus, the predicted properties provide evidence of the safety and efficacy of 7-methylguanine for humans.

## CONCLUSIONS

Despite the ability of DNA-damaging drugs to kill cancer cells, resistance to chemotherapy and drug toxicity remain serious problems. DNA repair systems involving PARP-1 and PARP-2 play an important role in the normal development of the organism, but in anticancer treatment with DNA-damaging agents these proteins may decrease the therapeutic effect. A nucleic acid metabolite 7-methylguanine was identified *in silico *as a novel inhibitor of PARP catalytic activity and studied experimentally. Two structural features of purine derivatives were shown to be important for efficient binding - the amino group at position 2 and the methyl group at position 7. At PARP inhibitory concentration, 7-methylguanine itself was not cytotoxic but able to sensitize BRCA1-deficient breast cancer cells to commonly used chemotherapeutic agents (cisplatin and doxorubicin). 7-Methylguanine is a nucleic acid metabolite observed in human serum and excreted in urine [52]. Despite the fact that 7-methylguanine is a weaker inhibitor than olaparib and some other PARP inhibitors, we believe that this natural compound possesses better pharmacokinetics and an adverse-effect profile compared to synthetic inhibitors and may be considered as a promising new constituent of anticancer therapy.

## Abbreviations

PARP: poly(ADP-ribose)polymerase
MD: molecular dynamics
